# Adult-type granulosa cell tumor of the testis: A case report and review of the literature

**DOI:** 10.1016/j.eucr.2023.102532

**Published:** 2023-08-16

**Authors:** Matthew Satariano, Emily Losinski, Jason Lane, John Wegryn

**Affiliations:** a Northeast Ohio Medical University, 4209 St. Rt. 44, PO Box 95, Rootstown, OH, 44272, United States; b Department of Urology, Cleveland Clinic Akron General, 1 Akron General Ave, Akron, OH, 44307, United States; c Department of Pathology, Cleveland Clinic Akron General, 1 Akron General Ave, Akron, OH, 44307, United States

**Keywords:** Granulosa cell tumor of the testis, Painless testicular swelling

## Abstract

Granulosa cell tumors (GCTs) of the testicle are a rare subtype of sex cord stromal tumors which usually present with painless testicular swelling. The histology of an adult testicular GCT often resembles normal granulosa cell tissue morphology, with similar immunohistochemical staining pattern such as positivity for steroidogenic factor 1 (SF1), calretinin, and inhibin A.^4,5^ We present a case of a 39-year-old male with an adult GCT of the testicle who underwent successful unilateral orchiectomy. This case adds to the body of literature and furthers our understanding of tumor histopathology, tumor behavior, clinical surveillance, and treatment strategies for this testicular neoplasm.

## Highlights

• Granulosa cell tumors are rarely found in the testis.
• Adult testicular GCTs are usually benign and involve painless testicular swelling.
• Adult testicular GCTs are typically treated with orchiectomy.

## 1 Introduction

Malignant testicular neoplasms are comprised of germ cell tumors (approximately 95%) and sex cord stromal tumors (approximately 5%). Sex cord stromal tumors are further sub-divided into Leydig cell tumors and Sertoli cell tumors which account for 1–2% and 0.1% of testicular neoplasms, respectively.^1^ Granulosa cell tumors (GCTs) of the testicle are a very rare subtype of sex cord stromal tumor which are classified as either juvenile (69%) or adult type (31%).^2^ The first adult GCT of the testis case was reported in 1952 with less than 100 reported cases since then.^1^ These neoplasms usually present with painless testicular swelling and rarely involve gynecomastia and sexual side effects such as decreased libido and erectile dysfunction.^3^ Adult GCTs of the testicle occur at a mean age of 40 years old and have malignant potential whereas juvenile GCTs of the testicle are benign and occur in the first 6 months of life.^4^ Although the majority of adult GCTs of the testicle are benign (∼84%), prophylactic orchiectomies are still used for treatment.^5^ Risk factors for metastasis include age of diagnosis over 50 years, tumor size >4 cm, rete testis or vascular invasion, and mitosis greater than 30 per 10 microscopic high power fields (HPF).^5^ Retroperitoneal lymph node dissection should be completed if there is suspicion for metastases. The histology of an adult testicular GCT often resembles normal granulosa cell tissue morphology, with similar immunohistochemical staining pattern such as positivity for steroidogenic factor 1 (SF1), calretinin, and inhibin A.^4^^,^^5^ Call-Exner bodies (microfollicular) and palisading patterns may also be present along with grooved nuclei. SALL4, OCT3/4, placental alkaline phosphate (PLAP), epithelial membrane antigen (EMA) and c-Kit/CD117 are negative for these neoplasms.^5^ It is important to note that immunohistochemistry is typically used to rule out other diagnoses. Furthermore, ultrasonography typically reveals solid hypoechoic well-circumscribed masses with an average tumor size around 2.2 cm^5^

## 2 Case report

**Fig. 1:**
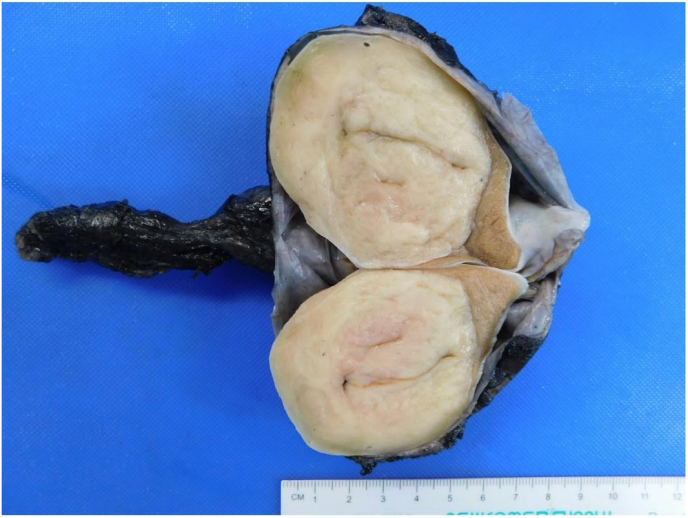
Gross examination of the testis revealed a tan-white mass of uniform appearance with no significant hemorrhage, necrosis or cystic degenerative changes.

**Fig. 2:**
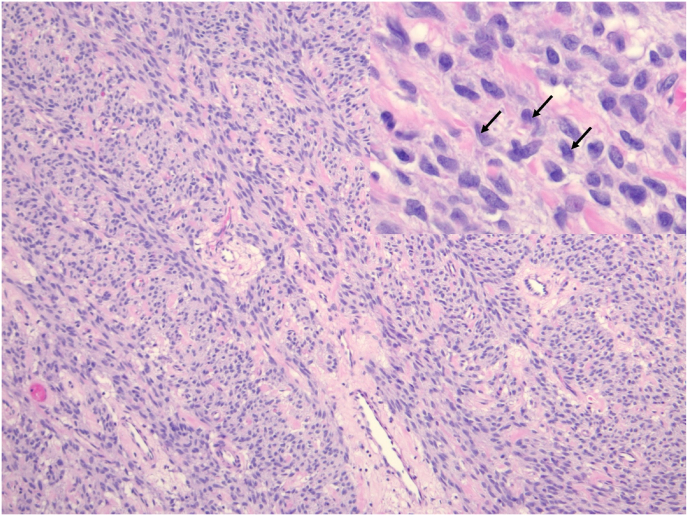
H&E stain, 100× total magnification. A diffuse growth pattern of tumor cells with predominantly spindle cell morphology was appreciated. At high power magnification (inset, 400×) tumor cells demonstrated moderate lightly eosinophilc cytoplasm and contained elongated nuclei, with rarely identified nuclear grooves (arrows).

**Fig. 3:**
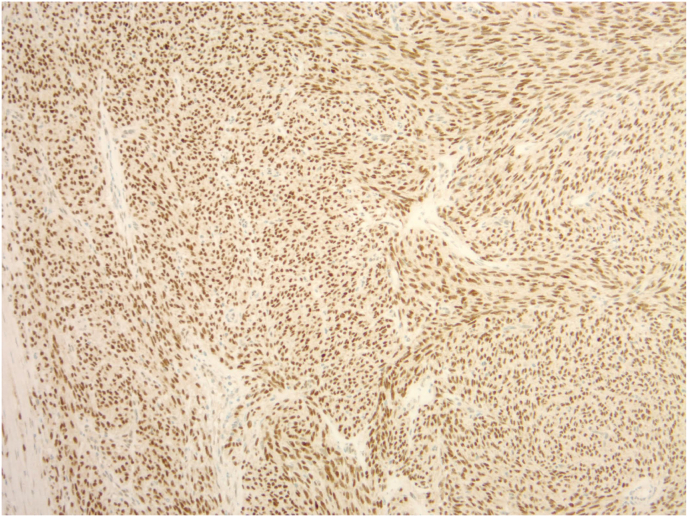
SF1 immunostain, 100× total magnification. The tumor cells showed strong and diffuse nuclear expression of SF1, which, along with the tumor morphology, offered strong adjunctive support for the diagnosis of GCT.

We present a case of a 39-year-old male with an adult granulosa cell tumor of the testicle. He had no significant past medical or surgical history and detailed a family history of breast cancer. He initially presented to urgent care with complaints of groin swelling which began two days prior. He did not have any other complaints and denied any genitourinary symptoms or trauma. Scrotal ultrasound was obtained which demonstrated a 6.1 × 5.1 × 4.5 cm well-circumscribed rounded, heterogeneous, hypoechoic mass involving the right testicle with marked increased vascularity as well as a large right hydrocele. He was subsequently evaluated in the urology office where on physical exam a large hydrocele was noted with an enlarged, firm right testicle. Laboratory markers including LDH, AFP, and HCG were obtained, which were all within reference range. He underwent an uncomplicated right inguinal radical orchiectomy. Gross pathologic examination of the resected specimen revealed a 5.2 cm tan-white mass of uniform appearance with no significant hemorrhage, necrosis, or cystic degenerative changes (Fig. 1). Representative hematoxylin and eosin (H&E) stained slides of the mass showed a diffuse nodular growth pattern comprised of cells with predominantly spindle cell morphology (Fig. 2). At high power magnification, the tumor cells showed moderate lightly eosinophilic cytoplasm and contained elongated nuclei, with rarely identified nuclear grooves (Fig. 2, inset). Given the unusual morphology, a broad immunohistochemical stain panel was applied, and the tumor stained positive for SF1, smooth muscle actin, S-100, and calretinin (Fig. 3) and was negative for myogenin, CK7, CD34, desmin, and EMA. The tumor morphology and immunohistochemical staining profile was consistent with an adult granulosa cell tumor. Surgical margins were negative for tumor and there was no evidence of angiolymphatic invasion. Computed tomography (CT) scans of the abdomen and pelvis as well as a chest x-ray were obtained post-operatively which did not demonstrate any evidence for metastatic disease. Additionally, following surgery, the patient was scheduled to have levels of LH, FSH, progesterone, estrogen, estradiol, and cortisol evaluated at several months post-operatively.

## 3 Discussion

Adult granulosa cell tumors of the testicle are a rare subtype of sex-cord stromal tumors usually presenting with painless testicular swelling. Our patient presented in the classic fashion. The only risk factor present for metastases was tumor size of 5.2 cm. Given that the patient lacked multiple risk factors for metastases, after a thorough discussion with the patient and multiple providers, observation with close surveillance was decided upon. The plan is to follow patient with imaging and lab work moving forward.

## 4 Conclusion

The literature for adult type testicular granulosa cell tumors is sparse with under 100 case reports/case series noted. This case adds to the body of literature and furthers our understanding of tumor histopathology, tumor behavior, clinical surveillance, and treatment strategies for this rare testicular neoplasm. A follow up to the case report 5 years post-operatively would provide additional information regarding the natural history and malignant potential of this tumor histopathology in the adult population.

## Funding

This research did not receive any specific grant from funding agencies in the public, commercial, or not-for-profit sectors.

## Consent

The proper consent was obtained for this research.

## Declaration of competing interest

There are no conflicts of interest.
